# A 9‐month‐old with wheezing and acute hypoxic respiratory failure

**DOI:** 10.1002/ccr3.2134

**Published:** 2019-04-07

**Authors:** Matthew Wong, Rajeev Bhatia

**Affiliations:** ^1^ Pulmonary Medicine Akron Children’s Hospital Akron Ohio

**Keywords:** foreign body aspiration, wheezing

## Abstract

Foreign body aspiration remains a diagnostic challenge, especially in an infant. While acute onset wheezing is highly suggestive of an aspiration, wheezing carries a broad differential diagnosis resulting in frequent misdiagnoses. Many cases present with a range of nonspecific findings and normal imaging, stressing the importance of a high index of suspicion.

## 1 CASE REPORT

**Figure 1:**
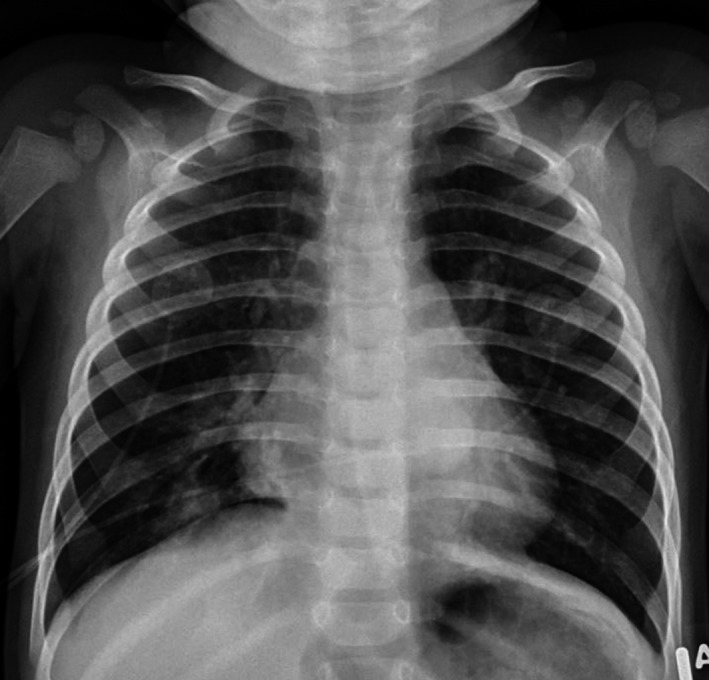
Frontal view chest X‐ray. Normal lung volumes with streaky opacities in both infra‐hilar regions concerning for early infiltrates

A 9‐month‐old full‐term unvaccinated Amish female baby with no known significant past medical history presented to the emergency department via EMS with fever, cough, and acute increased work of breathing. The patient was ill‐appearing in significant respiratory distress with bilateral wheezing on examination. There was no clinical improvement following a nebulized albuterol treatment, and she quickly required intubation secondary to persistent tachypnea. A chest X‐ray revealed bilateral infra‐hilar streaky opacities, worse on imaging immediately following intubation (Figure 1).

**Figure 2:**
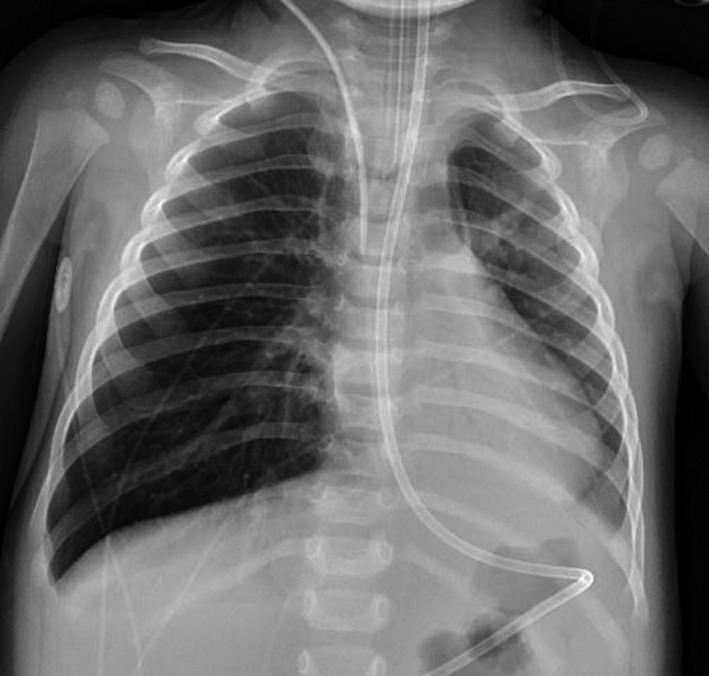
Frontal view chest X‐ray. Atelectasis of the left lung base. The right lung is clear with some over inflation relative to the left lung

A complete blood count was grossly unremarkable. She received a dose of ceftriaxone and was admitted to the pediatric intensive care unit, where she was continued on piperacillin/tazobactam (Zosyn). Notably, the patient had previously been hospitalized in the intensive care unit 21 days prior for acute hypoxic respiratory failure secondary to a left lower lobe pneumonia following an unwitnessed aspiration while being bottle‐fed. During this prior hospitalization, her initial chest X‐ray showed a right peri‐hilar opacity and tracheal aspirate culture was positive for both *Streptococcus pneumoniae* and *Haemophilus influenzae*. Clinical improvement was achieved with both bronchodilator therapy and a 7‐day course of ceftriaxone*.* However, following hospital discharge, parents reported minimal clinical improvement on scheduled albuterol with continued episodes of increased work of breathing, persistent coughing, and wheezing. She developed fever and acutely worsening respiratory symptoms, thus prompting this current presentation (Figure 2).

**Figure 3:**
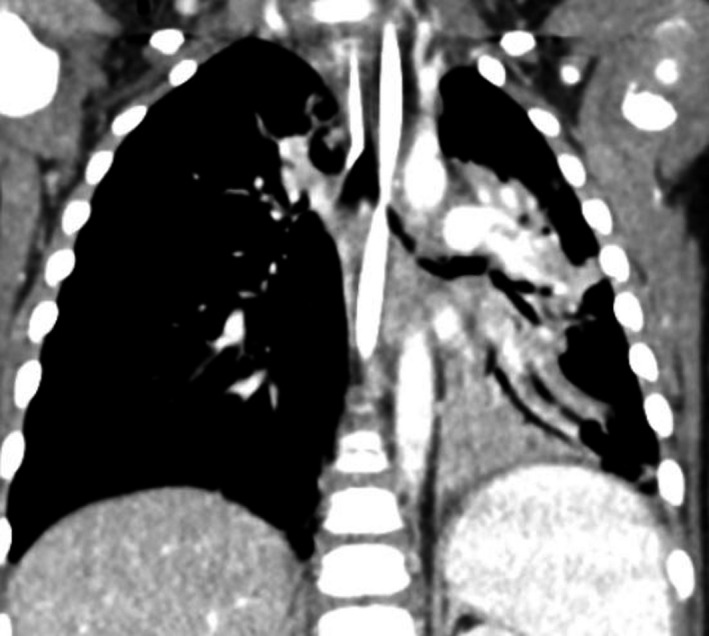
CT of chest with contrast. Overall radiographic interpretation identified narrowing of the left main stem bronchus containing a transverse dimension irregular filling defect. The left lung shows marked volume loss. There is also collapse of the left upper lobe segment. No vascular ring or sling is identified. In this section, you can also visualize significant left lower lobe involvement

Despite aggressive bronchodilator therapy and airway clearance, she continued to show clinical obstructive airway disease. Her tracheal aspirate culture was positive for *H influenzae*, while her respiratory viral panel detected both rhinovirus and enterovirus. Piperacillin/tazobactam (Zosyn) was discontinued 48 hours after a repeat tracheal culture showed no growth. Although her initial chest X‐ray showed acute left‐sided lung collapse, parents denied any choking episodes or the possibility of a foreign body aspiration (FBA) prior to symptom onset. Daily chest X‐rays were significant for persistent atelectasis, primarily of the left lower lobe (Figure 2). computed tomography (CT) of chest showed no anatomic ring or sling, but significant left lung volume loss and complete opacification of the left lower lobe (Figure 3). At that time, an irregular filling defect in the left main stem bronchus seen on CT was suspected to be secondary to secretions, debris, and inflammation rather than a foreign body.

**Figure 4:**
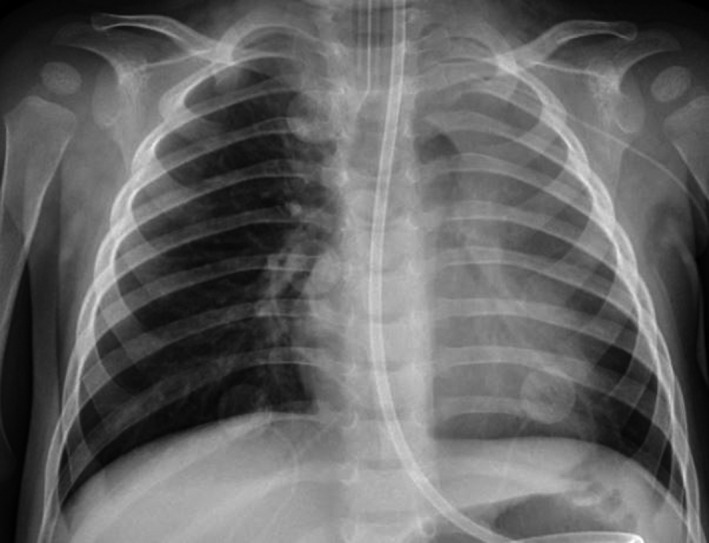
Frontal view chest X‐ray prior to the second bronchoscopy. This image shows significant left lung involvement, but of note are left upper lobe opacification and streaky peri‐hilar opacities

A bedside bronchoscopy demonstrated significant mucus obstruction from the proximal left main stem bronchus, requiring robust secretion retrieval from the left distal branching airways. Dornase alfa (Pulmozyme) was started to promote mucus clearance with subsequent chest X‐rays showing improved aeration of the left lung. She began to display signs of clinical improvement; however, she became septicemic delaying the plans for a repeat bronchoscopy. She was started on 48 hours of vancomycin and ceftriaxone with no organisms on repeated cultures taken after antibiotic initiation. Throughout her intensive care hospitalization, her respiratory examination was not consistently focal. Fluticasone propionate (Flovent) was started due to acutely worsening bilateral wheeze. A chest X‐ray showed return of left lung collapse, and a hospital‐acquired pneumonia was suspected. Due to multiple 48‐hour antibiotic courses, she was continued on 10 days of vancomycin and cefepime (Figure 4).

**Figure 5:**
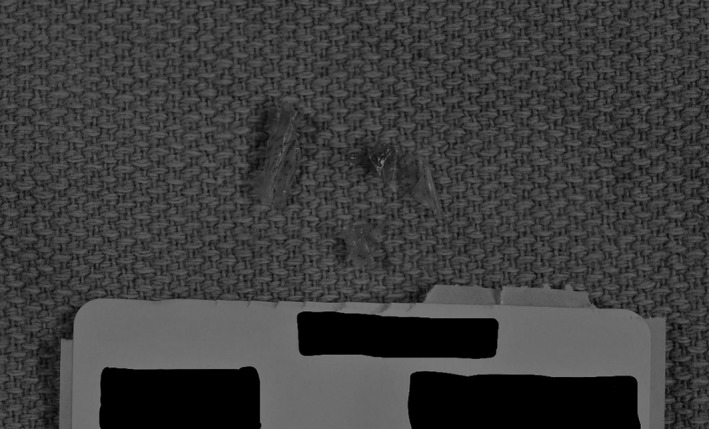
Three foreign bodies removed from the patient's lung

Once she became more clinically stable, a second bronchoscopy visualized transparent foreign body pieces in the left upper, right lower, and left lower lobes with segments adhering to the lung wall. Rigid bronchoscopy was successful in removing these foreign body pieces, which were suspected to be candy wrapper fragments (Figures 4, and 5).

At this point, parents reported that an older sibling had been feeding the patient candies prior to her initial previous hospitalization 1 month prior. Following the removal of these foreign body pieces, the patient had rapid and complete return to clinical baseline with no further respiratory issues prior to the hospital discharge.

## 2 DISCUSSION

Wheeze is a high‐pitched musical sound produced by turbulent airflow through narrow airways. Although asthma is a common cause of wheezing, infection and anatomic malformations of the airways are the major causes of wheezing in the infant population.^1^ Most cases of wheezing in children <3 years of age are transient and secondary to an infectious etiology, usually a viral respiratory infection such as respiratory syncytial virus bronchiolitis. Anatomic malformations should be considered in young infants presenting with wheeze: Congenital cardiac conditions resulting in pulmonary venous congestion can initially present as wheeze.^1^ Vascular rings/slings can obstruct airways causing wheezing, stridor, or respiratory distress.^1^ “Noisy breathing” associated with tracheobronchomalacia is often interpreted by caretakers as wheeze.^1^ The onset and duration of wheeze is also important to clarify. Chronic wheezing necessitates consideration of diseases such as cystic fibrosis, primary ciliary dyskinesia, and gastroesophageal reflux.^1^, ^2^, ^3^ In one instance, an infant presented with chronic wheezing and recurrent respiratory symptoms was discovered to have a congenital diaphragmatic hernia.^4^ Therefore, many different processes besides asthma may initially present with wheeze and it is important to create a broad differential during initial workup.

Foreign body aspiration is a life‐threatening pediatric issue with various initial presentations. It accounts for the 6th most common cause of accidental death in children and is the most common cause of unintentional injury in children <1 year of age.^5^, ^6^ While the exact rate of foreign body aspiration in infants <1 year is unavailable, 70% of all foreign body aspirations occur in children under the age of 3 years, with a peak incidence between 1 and 2 years of age.^7^ Infants are at the highest risk due to factors including a fondness for placing objects in the mouth, lack of teeth, and developing coordination with swallowing.^7^ The challenge with recognizing a foreign body aspiration is that presenting symptoms can vary depending on the location of the foreign body: Laryngeal lodging of a foreign body (common in infants) may cause stridor; local irritation and edema of a foreign body in the more distal bronchial tree cause wheezing and coughing.^1^ Due to the physical size of their chests, it is often hard to appreciate focal breath sounds in infants, which makes diagnosing a foreign body aspiration based on clinical findings even more challenging. A foreign body aspiration remained high on the differential for our patient, as she continued to display severe obstructive disease, in spite of aggressive intensive care therapy. This prompted the initial bedside bronchoscopy as well as obtaining the chest CT, in order to rule out the presence of a foreign body, as well as any anatomic malformation which could contribute to her difficulty weaning off high ventilatory support. Furthermore, we suspect that due to the size of her chest wall, it was difficult for providers to appreciate focality on her respiratory examination, as it is in any infant <12 months.

Unsurprisingly, delayed diagnoses of foreign body aspirations occur frequently and result in significant complications and increased mortality. Younger age, those from rural communities, and unwitnessed aspirations are more likely to have a delayed presentation.^7^ Our patient possessed these exact risk factors. It is believed that because children remain relatively asymptomatic immediately following an aspiration event, families attribute subsequent symptoms such as fever, coughing, and wheezing to other causes include respiratory illness.^7^ These children are often misdiagnosed with pneumonia or asthma, leading to unnecessary antibiotic or bronchodilator treatment.^8^ In a study of 76 children with a known foreign body aspiration, six patients presented secondary to recurrent respiratory infections, with three of these children symptomatic for greater than 3 months prior to the identification of a foreign body aspiration.^9^ Hence, the importance of both a high index of suspicion and a robust clinical examination cannot be further stressed. The typical presentation of choking followed by coughing, unilateral wheezing, or diminished breath sounds is present only in 40%‐57% of cases.^7^ Wheezing remains the most common physical examination finding (58%) and is independently associated with an increased odd of foreign body per bronchoscopy.^10^ However, as many as 30% of patients with a foreign body aspiration have no appreciable physical examination findings on the initial medical evaluation.^7^, ^11^

While plain radiography is the most common initial diagnostic test, it carries a low sensitivity for identifying foreign bodies as only about 10% of aspirated material is radiopaque.^6^, ^12^ A study of patients with aspirated foreign bodies removed by bronchoscopy showed that approximately 65% had normal radiographs.^13^ Air trapping was found to be the most sensitive and most specific finding, but it is not always present nor commented upon.^14^ Currently, rigid bronchoscopy is the definitive method for both the diagnosis and removal of an aspirated foreign body; however, CT scanning is a noninvasive alternative with both high sensitivity (98%) and specificity (97%).^15^ It is an excellent tool for suspected foreign body aspirations in the clinically stable child with inconclusive plain radiographs; indirect radiographic signs suggestive of a foreign body (air trapping, atelectasis, and collapse) can be accurately demonstrated.^5^ Additional benefit includes the ability to evaluate other differential diagnoses such as mucous plugs or the presence of anatomic anomalies.^6^ Nevertheless, bronchoscopy is indicated if clinical suspicion for a foreign body aspiration is elevated.^16^

## 3 CONCLUSION

The evaluation of wheezing in the infant population is a diagnostic challenge, requiring a broad differential diagnosis, an astute attention to detail, and a robust clinical examination. Although foreign body aspirations can initially present with a myriad of nonspecific findings, any child who presents with an acute onset of wheezing should be promptly evaluated for a possible foreign body aspiration. It is important to remember that radiographic images can be normal and do not exclude the presence of an aspirated foreign body. Direct visualization with bronchoscopy remains the definitive tool. Prompt recognition and removal of aspirated foreign material is imperative to avoid long‐term morbidity and prevent life‐threatening complications. Despite elevated concern for a foreign body aspiration, imaging and direct visualization of the airway was unable to identify the presence of multiple plastic foreign bodies in our patient. Furthermore, failure to achieve clinical improvement on aggressive bronchodilator therapy signaled the need to question continuing treatment while concurrently investigating other diagnoses. Therefore, our case serves as a necessary reminder of the importance in maintaining a high index of suspicion in any infant presenting with wheezing.

## CONFLICT OF INTEREST

There are no conflicts of interest to disclose.

## AUTHOR CONTRIBUTION

MW: wrote, edited, and revised the manuscript. RB: edited the manuscript and approved the final manuscript.
